# Problem-sustaining patterns: redesigning the concept of mental disorder

**DOI:** 10.3389/fpsyt.2025.1382915

**Published:** 2025-03-10

**Authors:** Sander A. Voerman, Derek W. Strijbos, Anton B. P. Staring, Femke de Boer, Matthijs van Dijk, Jim Driessen, Gerrit Glas, Rutger Goekoop, Annemarie Mulder, Nynke Tromp, Marloes Verhaar, David van den Berg

**Affiliations:** ^1^ Department of Clinical, Neuro- & Developmental Psychology, Vrije Universiteit Amsterdam, Amsterdam, Netherlands; ^2^ Mark van der Gaag Research Centre, Parnassia Academy, The Hague, Netherlands; ^3^ Faculty of Philosophy, Theology and Religion Studies, Radboud University, Nijmegen, Netherlands; ^4^ Center for Developmental Dsorders, Dimence Groep, Deventer, Netherlands; ^5^ Psychose Zorg, Psycholoog Nederland, Utrecht, Netherlands; ^6^ Reframing Studio, Amsterdam, Netherlands; ^7^ Faculty of Industrial Design Engineering, Delft University of Technology, Delft, Netherlands; ^8^ Department of Philosophy, Vrije Universiteit Amsterdam, Amsterdam, Netherlands; ^9^ Department of Research and Education, Geestelijke Gezondheidszorg Eindhoven, Eindhoven, Netherlands

**Keywords:** problem-sustainment, patterns, mental disorder, diagnosis, revisionism, agency

## Abstract

We propose the concept of a *problem-sustaining pattern* as a revision of the established concept of mental disorder. The proposed concept preserves valuable features of the established concept, such as recognition of the client’s hardships and scientifically informed justification of specific interventions. However, several assumptions behind the established concept have been widely criticized, both in terms of their clinical and moral normativity as well as their ontological and empirical soundness. We argue that a focus on problem-sustainment allows us to reframe the issue of demarcation in a way that helps avoid stigmatization while clarifying the role of client agency in diagnosis. We also propose a shift toward thinking in terms of patterns of dynamic interaction, which is more in line with current developments in complexity science. We conclude the article with a discussion of further research that would be needed to address various questions raised by our proposal.

## 1 Introduction

In this paper we propose the concept of a *problem-sustaining pattern* as a revision of the established concept of mental disorder^1^. The concept we propose emerged from collaboration between stakeholders, designers, and researchers in the field of mental health. It may be defined as follows:

problem-sustaining pattern:

a pattern of dynamic interaction between biological, psychological, and/or social factors that persistently or recurrently counteracts or undermines everyday problem-solving activities^2^.

Our proposal is revisionary in the sense that we aim to redesign the role of the concept of mental disorder in the practice of mental healthcare^3^. The need for such a revision derives from various scientific, philosophical, clinical, and ethical concerns. Our aim in this paper is to articulate these concerns and to explain how the concept of problem-sustaining patterns would help to address them.

The role that this concept would play in the practice we envision is sufficiently different from the established usage of the concept of mental disorder, that it would be impractical and confusing to use the term ‘mental disorder’ in all cases where it makes sense to talk about problem-sustaining patterns. Therefore, we propose to revise both the concept of mental disorders and the language of ‘mental disorders.’ Nevertheless, the point of this paper is not that mental disorders do not exist, or that we have no knowledge about psychopathology. Rather, it is to improve the concept in various ways, and to reframe our knowledge about it.

The structure of this paper is as follows. In section 2 we discuss the features and flaws of the established concept and the practice of psychiatric diagnosis. In section 3 we mention alternative approaches to mental healthcare that have departed from the established concept in various ways. Section 4 focuses on how the notion of *problem sustainment* differs clinically and ethically from the normative assumptions behind the established concept. Section 5 deals with how *patterns of dynamic interaction* differ ontologically and scientifically from the established concept of mental disorders. Finally, in section 6 we conclude the paper by summarizing our proposal and by formulating challenges for further research.

## 2 Features and flaws of the established concept of mental disorder

The ideas in this paper were developed within the *Redesigning Psychiatry* program (15, 16), a ‘design-driven program of activities’ ( (17), p. 124) within the Dutch mental healthcare system, since 2015. This program aims to develop models, narratives, methods, and technologies that are needed for long term transformative innovation in the mental healthcare sector^4^.

One of the most contentious issues we ran into over the course of this program has been whether or not to adopt a medical model of mental healthcare as the diagnosis and treatment of mental disorders. This issue arose during interviews, literature research, and design thinking workshops with service users, health professionals, experts by experience, designers, clinical researchers, managers, ethicists, and philosophers^5^.

There are various alternative approaches to mental healthcare innovation that depart from the established concept in different ways. Some approaches maintain the basic idea of diagnosis of disorders but reject the established systems of classification in favor of new frameworks. Other approaches rely on different models that are compatible with psychiatric diagnosis but do not require it. And some have explicitly rejected the very idea of mental disorders. In section 3 we will discuss some of these alternatives.

It might seem that the reasons for and against using the established concept, which we are about to discuss, lead to a kind of trade-off: the more one relies on the established concept, the more one stays vulnerable to its problems; the more one departs from it, the more one loses its benefits. But this only happens if we assume that the idea of mental disorder is a given, a fundamental notion that we cannot change. Instead, we have come to view the concept of mental disorder not merely as a concept to be analyzed in order to understand the domain that we are trying to design for, but also as one of the very ‘products and services’ that needs to be redesigned. From this point of view, the reasons for using the concept point to valuable *features* that many stakeholders require, while the reasons against it indicate a number of *design flaws* that have undesirable consequences.

In the workshops we organized, participants were therefore encouraged to approach the concept of mental disorders as an artifact with both features and flaws, something to be reshaped and reframed in view of how it mediates the human interactions that we seek to improve in the future of mental healthcare^6^. The most important *features* that participants mentioned include recognition, explanation, evidence-based treatment advice, access to specialized healthcare, sick leave, and social security. The most important *flaws* of the concept that participants mentioned were stigma, pathologizing of diversity, attribution of complex social problems to individuals, overmedicalization, iatrogenic effects, unethical power dynamics, invalidity of classification, and reification of syndromes. The purpose of this paper is to explain how our proposal might help to save the features while avoiding the flaws.

We will briefly discuss these features and flaws in the light of two overall areas of concern. The first involves *clinical and moral normativity*: what normative criterion or principle to distinguish between *healthy* and *unhealthy* is presupposed in the established concept, and what are its ethical ramifications for the duties and aims of mental healthcare? The second area of concern involves the *ontological and empirical soundness* of the established concept: what is the nature of mental disorders and how strong is the empirical evidence for the mental disorders that people are diagnosed with? The distinction between these two areas is not clear-cut, but it will help us organize the concerns that our proposal in this paper is meant to address. For the purposes of this paper, the first area of concerns precedes the second. After all, it is only if we believe that some people need mental health care and that society has a duty to provide it (clinical and moral normativity), that we have an interest in making sure that mental health care actually works (empirical and ontological soundness).

### 2.1 Considerations of clinical and moral normativity

One of the most important features of the concept of mental disorder is that we use it to justify certain forms of mental healthcare (21). Although there are also forms of mental healthcare that are accessible to anyone who applies for it, many countries reserve access and/or reimbursement of various interventions for those who have been diagnosed with the appropriate disorder. Furthermore, being diagnosed with a mental disorder can sometimes provide recognition of the hardships a person has been through, or of the additional efforts they have been making to cope with their environment. These practical ramifications are important normative features of the concept of mental disorders that many service users understandably want to preserve.

Despite these features, the concept of mental disorders is also widely understood as having severe flaws: it stigmatizes people (22, 23), pathologizes diversity by limiting the range of behaviors, customs, or needs that are accepted as normal in our society (24), and looks for individual dysfunctions when people are suffering from harmful social interactions. At the root of these controversies is a common suspicion that there is something fundamentally *arbitrary* about the normative assumptions behind our idea of mental disorders. This has at least been true historically, considering the kind of behaviors that have been classified as disorders in the past. Homosexuality, for example, was pathologized simply because it was viewed as abnormal by society’s standards (25). But these standards were arbitrary and harmful. Since then, the practice of diagnosing mental disorders has followed a kind of reflective equilibrium trajectory (26, 27), where general criteria and definitions of mental disorder were updated to reflect society’s changing intuitions about what is normal and functional behavior, and our understanding of various particular disorders and classifications were subsequently adjusted to reflect the evolving general criteria.

To some extent, this has reduced the harmfulness in the flaws of the concept. For example, in the Diagnostic and Statistical Manual of Mental Disorders (DSM), we have seen a growing emphasis on distress or disability as a criterion of mental disorder from the DSM-III onwards, such that behaviors that neither cause distress nor limit a person in how they want to live, no matter how uncommon or frowned-upon, are no longer the business of mental healthcare (28–30). Note, however, that the mere fact that a behavior is frowned upon by society typically already causes distress for a person and limits what they are able to do. This is perhaps why the DSM definition of mental disorder also mentions as a general criterion that ‘socially deviant behavior’ is insufficient in the absence of a ‘dysfunction in the individual’ (30). But it is questionable whether this really improves the situation. On the one hand, as we shall discuss below, the empirical validity of this assumption about dysfunctions is problematic. On the other hand, current classification systems like DSM-5-TR and the International Classification of Diseases, 11th Revision (ICD-11) (31) are still essentially lists of pathological symptoms, such as restricted interests and repetitive behavior (autism spectrum disorder), hearing voices (e.g. schizophrenia), or enjoying pain (sexual masochism disorder). This reinforces the stigma on those experiences both in cases where someone does and someone does not need mental healthcare.

Ultimately, the question how we can distinguish mental health from disorder has posed a philosophical problem—the *demarcation problem*—because it has so far seemed that neither biological, nor individual, nor societal criteria provide an adequate basis to distinguish mental health from disorder. We will discuss this debate in section 4.1.

### 2.2 Considerations of ontological and empirical soundness

Let us now turn to our second area of concern, involving ontological and empirical soundness. An important feature of the currently established practice of psychiatric diagnosis is the idea that we should not treat every mental health problem with the same, generic approach. Successive versions of the DSM and ICD have increased the reliability of the classification of mental disorders, which has been instrumental to the establishment of an evidence-based practice of mental healthcare. Instead of ideological disputes between therapeutic schools of thought on how to treat all mental health problems generically, we now have decades of research about the effects of various interventions in relation to different diagnostic classifications.

Regrettably, an important flaw of established disorder classifications is that they do not have the external validity that they were supposed to have (32–36), which means that our knowledge about the effectivity of the interventions themselves becomes difficult to apply in individual cases. This may not merely be a flaw in the current selection of symptoms for each syndrome in DSM-5 and ICD-11. Several critics have attributed the poor validity of established classifications to the methodological assumption that there *are* clusters of symptoms to be found *such that* for every cluster, the symptoms associated with that cluster are caused by an underlying (or latent) dysfunction (37–40).

This assumption plays a central role in established views of the concept of mental disorder. On Boorse’s view, for example, disorders are ‘internal states that depress a functional ability below species-typical levels (relative to age and sex)’ (41). On Wakefield’s view, disorders are ‘harmful dysfunctions’ (42) such that dysfunctions are necessary but not sufficient conditions for disorder. Specifically, the necessary condition is that an ‘internal mechanism is unable to perform one of its natural functions’ ((43), p. 152), where natural functions are understood as evolutionary adaptations. And according to DSM-5-TR, a mental disorder ‘reflects a dysfunction in the psychological, biological, or developmental processes underlying mental functioning’ (30).

While the search for underlying disease mechanisms of psychiatric syndromes is ongoing, a growing body of research strongly suggests that few if any of these syndromes will eventually be explained by a singular or well-demarcated set of dysfunction(s) at the biological or psychological level (33, 44–47). Instead, mental health issues typically involve complex interactions between social, psychological, and biological factors, none of which need to qualify as dysfunctions in their own right. Merely the unfortunate combination of several factors, which are relatively unproblematic on their own, may lead to a situation that requires help.

It is important not to pathologize contributing factors that are not instrinsically dysfunctional. Since the 19th century, for example, hearing voices has been viewed, both in Western society and clinical practice, as *ipso facto* something pathological, for example as an indicator, prodrome or symptom of schizophrenia or other mental disorders^7^. But it turns out that many people hear voices without having a problem (48–51), and some people who recovered from problems involving psychosis still hear voices, but no longer in an undesirable interaction with other factors (52, 53). Furthermore, by pathologizing this phenomenon in itself, we make life more difficult for people who hear voices, both those for whom it is and for whom it isn’t a problem (leading us back to the moral concern about stigma).

In addition to these empirical concerns about the established practice of psychiatric diagnosis and classification, various critics have argued that this practice involves a *reification* of mental disorders (54–56). Reification is a tendency to treat the syndromes that people are diagnosed with as real things or properties, so to speak, in ways that are unjustified. However, there is no singular fallacy that reification refers to,^8^ and there is ongoing philosophical disagreement about which ontological interpretation of diagnosis is justified. Here we shall discuss three forms of reification that in our view constitute flaws in the established practice, and that we mean to address in this paper.

The first form of reification is the assumption that there are *independent* psychiatric diseases behind the different psychiatric syndromes, even when a single person has been diagnosed with multiple syndromes at the same time. This form of reification builds on the assumption about underlying dysfunctions that we have just discussed. Thus, if someone is diagnosed with both Attention-Deficit Hyperactivity Disorder (ADHD) and Borderline Personality Disorder (BPD), then it is often thought that this person really has two separate psychiatric diseases. However, if no corresponding singular underlying dysfunctions are to be found for these syndromes, as we discussed above, then given the fact that the definitions of these different syndromes largely *overlap*, it seems plausible to assume that saying that someone has BPD and saying that someone has ADHD amounts *partly* to the same thing (32, 59).

The second form of reification is the assumption that a mental disorder is something a person either does or does not have, like a viral infection or a broken bone. Psychiatric diagnosis is then understood as an attempt to establish whether someone has a disorder or not. Even when matters of degree are built into a classification, as in the case of Autism Spectrum Disorder (ASD), our healthcare system still requires that we establish whether or not someone has this disorder, and lay people often talk about people who are ‘on the spectrum.’ However, the reality of mental health is largely gradual, and nature provides no clear demarcation lines between healthy and disordered development, or between sadness and depression. We may sometimes have to draw such lines for clinical reasons, but that does not mean we should project those boundaries into our ontology of mental health.

The third form of reification is a confusion of the idealized constructs or models that we use to explain mental health problems with the actual mechanisms behind those problems in individual situations. Suppose that a person receives a BPD diagnosis even though the facts about their case left the choice between BPD and certain other syndromes largely undetermined. Once the diagnosis has been provided, though, both their practitioner and they themselves might be inclined to explain as much of their behavior as possible in terms of an unstable self-image, which is a prominent aspect of BPD. Even though self-image is a psychological construct, the instability of it is now being treated as a fact in their case. However, this might not always provide the best explanation, and were they to have received a Bipolar II diagnosis instead, for example, then perhaps a different type of explanation might have been applicable to a similar degree. But the idea of being diagnosed with a particular mental disorder seems difficult to square with the notion that various different idealized explanatory models might all be somewhat applicable to one’s individual life.

Perhaps it could be argued that the established concept of mental disorders can be maintained as long as one avoids these forms of reification by adopting a sufficiently constructivist or anti-realist attitude toward diagnosis. However, it seems to us that diagnosis should have *some* ontological upshot. First of all, it seems part of the very idea of diagnosis that there should be something for diagnosis to *get right*. Clients seek advice of experts to help them figure out *what is going on*. They do not merely want a story that sounds convincing or that would explain their symptoms if it were true. No, they want and deserve a hypothesis that is plausible based upon the information about their situation and the scientific knowledge about mental health problems. Of course, that knowledge is imperfect in many ways, and the practitioner should communicate the extent and limitations of the reasons for believing in the plausibility of the diagnosis. Second, a diagnosis should also inform treatment choices on the basis of facts and evidence. Ideally, a plausible analysis of a problem-sustaining pattern should also provide plausible clues of the ways in which such a pattern might be broken. But how could that possibly be the case if the attributed problem-sustaining pattern has nothing to do with what is going on in the real world? Finally, even in an imperfect system of diagnosis that is only facts-based in limited ways, it may still be possible for a practitioner to really *get it wrong*. To miss crucial symptoms, to postulate the wrong mechanism, and to recommend a therapy that will not work, while there were good reasons to recommend another intervention that likely would have helped. It seems hard to explain how this could be the case on a fully constructivist account of diagnosis.

At the same time, we recognize that diagnosis does have certain constructive aspects. There are many features of the models we use to recognize patterns that we should not read back into the real world. And being diagnosed in a certain way, and being recommended a certain intervention, are themselves psychological events that may influence a person’s experience of their problems and the result of the intervention. So the facts that are diagnosed may not be fully independent of the acts of diagnosis. In section 5 we will discuss these matters in further detail. We will then argue for an *interpretivist* account of pattern recognition, which combines constructive aspects of interpretation with a sufficient amount of realism about the patterns that may be captured by such interpretation. Our aim will be to provide a plausible ontology of mental health without the above three forms of reification.

## 3 Alternative approaches

There are many approaches to mental healthcare that have adopted alternative concepts or frameworks in addition to or as a replacement for the established classifications from DSM and ICD. In this section we will discuss the alternatives that have been influential in the development of our proposal.

### 3.1 New paradigms in nosology

The aforementioned concerns about the validity of the established classification systems of DSM-5 and ICD-11 have led to alternative proposals that seek to unravel the underlying causal mechanisms in a ‘transdiagnostic’ way, i.e., without being hindered by our current diagnostic categories. The most well-known are the *Hierarchical Taxonomy of Psychopathology* (HiTOP) (36, 60, 61) and the *Research Domain Criteria* (RDoC) (62, 63). These frameworks are dimensional instead of categorical: mental health problems are typically understood in a multifactorial manner, and the presence of each factor is gradual. The ontology of patterns that we are going to propose in section 5 will be similar in both respects.

However, in the frameworks of HiTOP and RDoC, these various factors are still understood to be intrinsically dysfunctional, rather than focusing on the interaction between factors to explain the sustainment of problems. Thus, in the HiTOP model, there is a large list of ‘maladaptive traits’ which are then clustered hierarchically into spectra and subfactors that are also considered maladaptive. The HiTOP model ‘presumes that each construct articulated in the model is undergirded by a disturbance in an essential psychological function,’ where such a disturbance ‘is defined by the HiTOP model as the failure of a psychological mechanism to perform the adaptive function for which it evolved’ [(61), p. 4]. And according to the RDoC proposal, ‘mental disorders can be addressed as disorders of brain circuits’ where ‘the dysfunction in neural circuits can be identified with the tools of clinical neuroscience’ (62).

Thus, HiTOP and RDoC fail to appreciate that it is better not to pathologize factors that only contribute to the sustainment of a problem in particular contexts and in interaction with specific other factors. Furthermore, both approaches assume that something can only be a health problem if its problematic nature can be reduced to an evolutionary or neurological dysfunction. Instead, we believe there is a better way to understand the normativity of mental health, as we will argue in section 4.

### 3.2 Holistic approaches

There are several approaches that do focus on the interaction between multiple factors as the key to understanding mental health issues. These include ‘holistic theory’ and ‘case formulation’ methods of cognitive behavior therapy (1, 64–66), complex systems approaches (40, 67–73), network theory (37, 38, 44, 74–76), ecological approaches (77), and enactivist approaches (78–81). What these approaches show is that the interrelations between factors are often organized in a nonlinear network, such that feedback loops and equilibria emerge. Our own proposal draws heavily from several of these approaches, and we shall refer to some of them in passing in the sections below.

### 3.3 Recovery and positive health

Perhaps one of the most influential alternatives to the medical model, the *recovery model* (82) emphasizes empowerment, peer-support, and rehabilitation (83–85). It does rely on the concept of a crisis—that from which recovery is possible—which may involve mental illness. Nevertheless, the insights and tools from the recovery approach are largely generic rather than disorder-specific, and the model is often used to develop alternative care practices which do not presuppose the concept of mental disorder (86). In contrast to the aforementioned approaches that have sought to explain the normativity of mental health in biological, evolutionary terms, the recovery approach sees the aims of recovery as highly personal and existential. The model of agency that we adopt as part of our account of problem-sustainment in section 4.2 bears some resemblance to this existential perspective.

A more recent alternative is the concept of *positive health*, which has been defined as ‘the ability to adapt and self-manage in the face of social, physical, and emotional challenges’ (87). While these challenges may include mental disorders, the purpose of the positive health approach is to shift the focus from curing disease toward increasing health. That involves both finding ways to live with illness as well as improving aspects of life that fit poorly into the medical model in the first place, such as financial stability or sexual satisfaction. The Institute for Positive Health uses a brief questionnaire to assess six dimension of health (including mental well-being) as a dialogue tool for practitioner and client to discuss which things the client would like to improve.

We think the tools and insights brought by the recovery and positive health approaches are mostly complementary to the practice of diagnosis. Both approaches help to broaden our perspective on health and healthcare and stimulate us to focus on factors beyond the medical model. However, neither the recovery model nor the positive health assessment is meant to capture our existing knowledge about the efficacy of various psychological and psychiatric interventions. When a person is stuck in a pattern that sustains their problem, it is desirable to know which intervention would be most likely to help break that pattern. In such cases, a diagnostic approach is needed, possibly in the context of a more overall plan of personal recovery or improvement of positive health.

### 3.4 Eliminativism

Some critics of the practice of psychiatry have argued that mental disorders do not exist and that the concept should be abolished. Historically, such criticism should often be understood in the context of a struggle for freedom and against repressive practices of institutional psychiatry [e.g. (88)]. More recent work in critical psychiatry has questioned whether psychiatric diagnosis is the appropriate starting point for mental healthcare (89–92).

The extent to which such approaches lose the features together with the flaws of the established concept depends largely on what alternative they envision. Thomas Szasz, for example, argued on libertarian grounds that mental disorders do not exist, which both meant that people could not be involuntarily treated, but also, in his view, that people did not have to be reimbursed for voluntary psychiatry (88). Reimbursement was a flaw from his point of view, while it is a feature in our view. A rather different form of eliminativism is that of Lucy Johnstone, who has argued against psychiatric diagnosis and the attribution of mental disorders, and who promotes psychological formulation as a better starting point for mental healthcare (65, 91). Our proposal in this article has much in common with psychological formulation, but we consider our proposal to be a form of diagnosis. In this case, the distinction between eliminativism and revisionism is to some extent verbal.

What matters for our purposes in this article is that there are important features of the established concept of mental disorders that we want to keep, such as reimbursement and the means to quantify and measure the effects of specialized interventions.

## 4 Problem-sustainment

In this section we shall articulate the ‘problem-sustainment’ part of the concept of problem-sustaining patterns. This notion is mostly intended to address the considerations of clinical and moral normativity, which we identified in section 2.1. In section 5 we will elaborate on the ‘patterns’ part of the proposal, which mostly involves the considerations of ontological and empirical soundness from section 2.2. Nevertheless, all of these matters are closely interrelated.

Recall our proposed definition of a problem-sustaining pattern: a pattern of dynamic interaction between biological, psychological, and/or social factors that persistently or recurrently counteracts or undermines everyday problem-solving activities. Problem-sustainment, then, is the persistent or recurrent counteracting or undermining of everyday problem-solving activities. What does this mean, exactly, and why would this be a useful notion?

In section 4.1 we will first introduce the overall idea in the context of the demarcation problem. After that we will examine the details of the proposed definition. Section 4.2 is focused on the notion of a *problem* and what we mean by everyday problem-solving. In section 4.3 we articulate the notion of *sustainment* by discussing the ways in which such problem-solving can be undermined. Finally, in section 4.4 we summarize how the combination of these notions is meant to address the aforementioned considerations regarding clinical and ethical normativity.

### 4.1 What is the source of clinical normativity?

At the end of section 2.1 we mentioned the demarcation problem: the problem of how to justify the normative distinction between mental health and disorder. There is a longstanding debate in the philosophy of psychiatry between *naturalists* and *normativists* about the grounds on which this distinction can be justified. Naturalists argue that the same empirical facts that explain the causes behind those behaviors or symptoms that we regard as mental illness must also justify *why* we regard them as mental illness. The view of Boorse that judgments about mental disorders can be reduced to facts about biological dysfunctions (41), which we discussed in section 2 above, is an example of this. Normativists, on the other hand, argue that empirical knowledge of how a state of affairs is caused or sustained, or how it might be changed, is different from justifying *whether* a state of affairs is a health issue, and how it *should* be changed. Within the normativist camp, some choose to focus on the social, cultural and political dynamics lying at the heart of the concept of mental illness. This is also known as social constructivism (93, 94). Others put forward accounts of health and illness in terms of (impediments to) individual agency and elucidate the inherent value-ladenness of this concept. We might term this ‘agential normativism’ (95, 96).

The problem of demarcation is that at face value, none of the sources of clinical normativity that any of these approaches provide seem appropriate in order to justify the claim that someone has a mental disorder or that they need mental healthcare. For example, naturalists often rely on an evolutionary concept of function as an objective criterion, but in a case where a person is having problems as a result of their impulsive behavior, why should it be relevant whether that behavior has been selected for by the process of evolution? At the same time, if demarcation could be justified on the individual agent’s terms alone, what would still be the role of the expert, and how would we deal with cases where people do not understand they have a mental health problem? Finally, if we accept the influence of societal values on clinical norms as a justification of those norms, then demarcation becomes a matter of historical and cultural contingency. Of course, proponents of each of these approaches are typically aware of these challenges, and try to address them. Our purpose in this article is not to criticize any of these approaches in the amount of detail that a fair critique would require. Rather, it is to explain how our proposal is meant to solve the overall problem, and how our solution may be compared to each of these existing approaches.

The solution we propose, simply put, is that people need mental healthcare when they have problems that are being sustained by the kind of patterns that fall in the domain of mental healthcare. At first, this formulation may sound trivial or circular, but it captures an important conceptual shift from demarcating the problems themselves to demarcating the *mechanisms of sustainment*. Theoretically, any problem that a person has can, but need not, be a mental health problem, depending on whether there is a pattern that prevents this person from solving it on their own. This does not mean that anything can be a problem, or that something only counts as a problem if the client thinks it does. Our proposal requires a comprehensive theory of what problems are, and we will sketch one in the next section.

Nevertheless, provided that something *is* a problem, we propose that it requires an intervention if and only if the problem-solving activities of the person or people in question are thwarted by a pattern of bio-psycho-social interaction that they cannot seem to break out of without the aid of mental healthcare. If someone is sad and lacks motivation to go to work or to enjoy life, then one way of dealing with that is to call in sick and stay at home. If that happens incidentally, or after a tragic event, for example, then this might be the right way to deal with it, to give oneself some space and time in order to recover, to process what needs processing, and to find that joy and motivation will return. On the other hand, if joy and motivation do not return, and if the sadness is related to low expectations of happiness or success that are enabled and sustained by the choice to stay at home and do nothing, then someone’s response to their problem might have become part of a pattern, along with other factors, that reinforces the problem.

We can make a distinction between (a) the problem that one faces and (b) the pattern that sustains the problem and (therefore) might become part of the problem. The advantage of our proposal is that we do not have to demarcate with respect to (a). That is: we do not have to distinguish pathological problems from non-pathological problems. For example, we do not have to distinguish between healthy and pathological sadness, or to determine how many weeks exactly it is still normal or healthy to be sad after a certain tragic event. Whether or not a problem is in need of professional care depends on (b), the presence of a pattern that sustain the problem despite attempts to solve it.

This does not mean that pragmatic criteria concerning the duration or severity of symptoms cannot be useful in the practice of diagnosis. Of course it is relevant to know whether someone has been sad for a week or a year. However, the established concept of mental disorders has resulted in extensive debates about what the right standards of normality are for phenomena like sadness (97) and bereavement (98). The latest editions of ICD and DSM now specify a criterion for prolonged grief disorder of persisting for 6 and 12 months, respectively. These standards are clearly arbitrary, and mostly reflect a kind of consensus among the authors on how to prevent ‘false epidemics’ (99–101). By contrast, we argue that what matters is whether a person’s manner of dealing with their problem is productive or not. Is the person processing their grief, or have they become trapped by it? Now suppose that this person has lost their child, then obviously we are not going to be asking this question after one week. But that is because grieving the loss of one’s child is such an immense process that a week is empirically the wrong timescale to establish whether there might be a problem-sustaining pattern undermining the grieving process.

So what is the source of clinical normativity on this view? Philosophically, the notion of problem-sustainment is meant to *divide* the normative challenge of justifying why people need mental healthcare into two components. First, the concept of a *problem* should explain why there is a normative reason to address that problem, but merely in a general sense that includes everyday problems and does not need to establish anything specific about health, clinical practice, or the need for professional assistance. To explain what problems are, in this sense, merely requires a *general* theory of human agency and problem-solving. Second, the concept of *sustainment* should explain when people are unable to deal with their own problems without the aid of (professional) care.

The first component may therefore be compared to the agential normativist approach mentioned above. As we shall see, something can only be a problem for an agent in relation to that agent’s values and goals. However, because this is only the first step of the analysis, we do not have to explain in terms of those values and goals whether someone needs help, we merely need to explain what it means for someone to have a problem. Furthermore, as we shall argue in the next section, their own values and goals may also be something that a person needs better understanding of through interpretation and exploration, which may be one of the problems they need help for. Therefore, the agential aspect of our proposal does not imply that the client could never be mistaken about whether they need help, what kind, or how much. Finally, the language and concepts we use for this process of self-interpretation reflect cultural values and ideas that invariably shape us, though they can also become the subject of reflection and criticism. Hence, the social values that constructivists allude to are not intrinsically valid, but they have an important role to play in the ways we understand ourselves and each other.

The second component complements the agential aspect with (a) science about how and to what extent people can get stuck in problem-sustaining patterns and (b) an account of the normative considerations that undergird the social contract that determines who deserves professional care (102). Ad (a), the patterns that sustain problems are patterns of biological, psychological, and social interaction, which may or may not be understood in naturalist terms^9^. However, these patterns do not establish substantial normative criteria for health or pathology, they merely explain when people are unable to solve their own problems without professional help. Ad (b), whether that help should also be provided is a further, societal question that is not established on scientific grounds only, but on a plurality of normative views and considerations that undergird relations between stakeholders in the social contract that underlies healthcare. As we shall see later on, patterns that sustain problems exist in degrees: sometimes people fail to fully solve a problem on their own, but the degree to which it keeps hindering them, or the frequency with which the problem keeps coming back, is perhaps manageable, or a cost that society is willing to accept in the light of scarce healthcare resources. Thus, when it comes to the question of who deserves care, as opposed to who could use help, there are trade-offs that demand political decision making. We will elaborate on these matters in the next few sections.

### 4.2 Everyday problem-solving

According to our version of the agential approach, it does not really matter how common or uncommon the problems in question are, or how normal or abnormal they might seem from the perspective of societal standards, to determine whether someone needs mental healthcare. Instead, according to our definition of problem-sustaining patterns, what matters is whether there is a pattern that *persistently or recurrently counteracts or undermines* a person’s attempts to solve their problem.

The notion of a *problem* in this definition is meant to include common problems. ‘Problems in living,’ to borrow a phrase from Thomas Szasz (88), that we all face every day. Let’s take a rather simple problem. You want to unlock your bike, but you cannot find your keys. Problem! Where did you leave your keys? If this happens incidentally, you will lose time, but you can probably solve this yourself. If this happens more often, then perhaps you need to find a more structural solution: perhaps you need to attach your bike key to your house keys, or rethink the place where you typically leave those keys. But for some people, even such solutions are inadequate, and their lives keep being disrupted by their tendency to lose and forget stuff. This is a common criterion for ADHD (103), and at least for some people, professional help, such as psycho-education, medication and/or psychotherapy can radically change how often this happens to them (104). At the same time, various social and environmental factors might influence how distracted a person gets, and after certain events this tendency to lose and forget things might be increased.

But the *problem* is still the everyday problem of not being able to unlock your bike when you cannot find your keys. There are no mysteries about why that is a problem. It is just that in some cases, there is a pattern of interactions, which in this example might include both neurobiological, psychological and social/environmental factors, that *undermines* a person’s attempts to solve the problem, and which someone might not understand or be able to control without diagnosis and evidence-informed intervention.

Of course, forgetting keys is unlikely to be their only problem. We can have knowledge of what other sort of problems people with similar neurocognitive variations might be typically having trouble to address^10^. Perhaps the person in our example will also have trouble keeping track of time, and no matter how many watches and clocks and alarms they use, keep missing appointments because of that. But the problem of missing an appointment is still an everyday problem, and once again there is no mystery about why this is a problem. What matters is that there might be a pattern that makes it hard for this person to prevent missing appointments all the time.

To be sure, not all problems are common. Sometimes people have uncommon problems and sometimes patterns sustain uncommon problems. The point of our proposal is that it does not matter how common or uncommon a problem is for the question of demarcation—what matters is whether there is a pattern that sustains the problem.

Nevertheless, even people who have less prevalent experiences, such as hearing distressing voices and being extremely preoccupied with the idea that they are followed and observed wherever they go, often end up having problems that can be typically understood in terms of values and needs that many people can relate to (106). If these experiences make it difficult to maintain relationships or pleasant contact with friends and family, if they result in losing one’s job, and if the response of society and/or health care is to isolate such a person from everyday life, then the problem is typically that the person in question wants to be able to do and have those things again, but is unable to, in addition to no longer suffer from the unpleasant experiences themselves^11^.

Therefore, in order to make our concept of problem-sustainment work, we do not need a theory of pathological problems. On the contrary, we need a theory of problems in general, problems that people are typically able to solve, unless there is a specific pattern that undermines their problem-solving activity.

One important aspect of human behavior that is crucial to mental healthcare is how much of our behavior, healthy and otherwise, is habitual, automated, embodied-embedded interaction with our environment and the people around us (80, 107). And our habits are guided and shaped by the social practices that we are part of, which to some extent embody our values and principles (12). At a first approximation, a *problem* is what happens when habitual behavior in a certain context, at some level, fails. This includes cases where it fails to fulfill its immediate purpose, such as when the simple habit of reaching in your coat pocket to get your keys leads to the surprise of finding that pocket empty. But it also includes cases where habitual behavior fails in some more general sense, such as when it fails to address the needs of a person who is feeling increasingly estranged while ostensibly going about their business successfully.

A specific type of problem that is of particular interest to healthcare is that of suffering. Many of the unpleasant sensations that people can suffer from are typically disruptive signals that interrupt our habitual behavior as a result of a special circumstance. Sometimes the problem is that we need to suddenly respond to something that could hurt us, or already is hurting us. Sometimes the problem is a physical or psychological wound that takes time to heal. While pain can be functional, prolonged suffering is something that people typically do not value, which makes it a problem. Hence, our proposed demarcation in terms of problem-sustainment also applies to cases of suffering: if there is a pattern of dynamic interaction that sustains the suffering in a manner that the person is not able to break without professional help, then they need help. Nevertheless, there are many cases where people are able to deal successfully with the causes of their suffering without mental healthcare.

Everyday problem-solving, then, is the process of managing our day-to-day agency, so to speak, in order to make sure we get back on track when our habits, in combination with our circumstances, lead us off-track. We can think of the activity of problem-solving in terms of a hierarchy of *cycles* of agency *on top of* the perception-action loops of habitual behavior. The most basic, everyday cycle of problem-solving is where the overarching habits don’t change, except perhaps for the degree to which they are getting more nuanced or attuned to the specific context as we get more practiced over time. We act habitually, until an exception comes up, which we address more attentively and manually, so to speak, after which we can return to acting habitually.

The second cycle is where our environment changes in such a way that we have to *change our habits* in order to be able to keep acting habitually. Maybe one way of achieving something doesn’t work anymore, and we have to solve the problem by finding another way.

Finally, the third cycle is when we have to change our habits because we are *revising our values and goals*. For example, a person may have come to realize that they are part of an unfair practice, or that some of their habits are sexist in ways they did not realize before. This may be prompted by a novel situation in which they are surprised by their own emotional response, or it may be that they have learned from others to look at familiar situations in a new way (108). In order to solve this problem, they will have to adjust their habits in such a way that they address their new moral concerns and put into practice their updated values.

This last cycle involves the existential dimension of our presence in the world: we are not merely beings driven by goals, but human selves facing the problem of what final ends or values to endorse or identify with (109–111). Such existential problems are of course not everyday problems in the sense that we do not question our final ends every day, but they are nevertheless a fundamental part of the human condition.

Furthermore, the cycle of value revision may operate over long periods of time, accumulating reasons, insights, or frictions as one’s reasons for changing one’s normative outlook slowly build up toward the point where more explicit reflection becomes necessary. Moreover, even though we can distinguish these three cycles conceptually, they are not separate activities. It is while attending to an exception that someone may realize this is happening too often and they need to adjust their habits. And it may be while going through the cycle of adjusting their habits to pursue their goals in a new environment that they start to realize that, perhaps, these are no longer the goals they want to pursue. In that sense, each cycle is part of our everyday problem-solving activity.

### 4.3 Patterns that prevent people from solving their problems

In terms of these cycles of agency, our proposal is that for each type of cycle, it is not how pathological or dysfunctional a problem is, but rather whether that cycle fails to address that problem, that determines the need for help. Note also that for each type of cycle, factors that undermine the cycle may be found at the biological, psychological and social levels. For example, the existential cycle can be undermined by social mechanisms that discourage critical reflection, but also by the psychological mechanism of confirmation bias, or by the biological effects of stress on the activity in one’s prefrontal cortex, which may severely affect one’s capacity to reason about goals (112–114).

Our proposed definition of ‘persistently or recurrently counteracting or undermining everyday problem-solving activities’ is meant to generically capture this range of different problem-sustaining patterns. In this section, we shall discuss some examples of these different types of patterns that sustain problems in various ways and at various levels.

For starters, let us briefly return to the example from the previous section of the person who is frequently losing their keys. In this case, they may in some sense be able to solve their problems on separate occasions, but because doing so takes so much effort that it still disrupts their lives, without addressing the underlying pattern, we can still say that their *everyday problem-solving activities are being undermined* by a pattern that they cannot address without professional help. The reason is not that they do not know what to do when they lose their keys, but rather that there may be underlying processes, perhaps of both a neurobiological (e.g., variation in dopamine and norepinephrine mediated reinforcement signaling (115)), psychological (ruminating a lot about loss, negative self-evaluation), or social nature (lifestyle norms and stigmatization in society, an extremely busy period at work, etc.), that cause the cards to be stacked against them.

In other cases, a person’s problem-solving activities may actually be *part* of the pattern. Consider sleeping problems. What is the problem? Lack of sleep. Why is it a problem? Because when you do not sleep enough, you are unable to function properly. Once again, this is not a special or pathological problem: everybody has lack of sleep sometimes, and everybody knows how this can affect you during the day. Nevertheless, if this happens more often, you might try to get more sleep by sleeping in until you have reached the amount of time you think you need. For some people, this can actually turn an incidental sleep problem into a structural problem because it disrupts various cycles that determine when you get sleepy in the evening (116). The reason that justifies mental healthcare in such a case is that a person is trying to solve their problem in a counterproductive manner, and that they need professional, evidence-based advice about how they can shift from this counterproductive equilibrium back to a healthy sleeping pattern.

Note how the counterproductive response to sleep deprivation involves a feedback loop between the problem and behavior in response to that problem, which then reproduces the problem. This is a typical example of what Bakker calls a ‘problem-maintaining circle’ (1, 64): a circular interaction between experiences and behavior that can be analyzed together with the client using a functional analysis or case formulation in cognitive-behavior therapy. Other examples include inactivity cycles: when you do less, you become less fit, which undermines your ability to do more, and causes you to do even less. But also: when you are afraid to try things or unmotivated to go out, you stay at home, and therefore will not have the experience of success or satisfaction when you do try things or go out, and therefore keep staying at home.

In Bakker’s view, the discipline of clinical psychology should develop its own practice of diagnosis, based on an inventory of these kind of circles that we know about from cognitive behavior therapy (1, p. 10; see also 65). He believes this should be an essentialist psychological framework, with the purpose of unearthing real psychological properties, while leaving any neurobiological aspects to psychiatric diagnosis. While we agree that case formulation should become a part of diagnosis, we do not rely on an essentialist conception of psychological properties. Rather, we will propose in section 5 that the factors in a case formulation or holistic theory are simplifications of fuzzy and gradual patterns, and we shall argue that this type of analysis should be unified with complexity models and neurobiological knowledge from psychiatry and neuroscience, in order to understand multilevel interactions, where social context, psychological experience and behavior, and neurobiological factors can all be understood to influence each other.

In the example of the person losing their keys and missing appointments, the interaction might include their behavior, their experience of time, functioning of their prefrontal cortex, their circadian rhythm, and various other factors. Furthermore, *how much* this disrupts their life also depends on the context. How often do they have appointments in the first place, and how complex and demanding are their career choices in terms of day-to-day scheduling and organization? In some cases, changes in the work environment might be preferable to medication, or it might be desirable to try both. This shows how social, psychological, and biological factors can interact with each other in a problem-sustaining pattern.

The concept of problem-sustainment is also meant to cover ways in which issues may remain *opaque* to the people affected by those issues. First of all, patterns that sustain problems are often difficult to discern because they are *diachronic*, which means that you have to collect pieces of the puzzle over time in order to even have the information that may or may not feature the pattern. If you repeatedly run into the same problem, but you usually manage to do something about it, then it may take a long while before you realize that you are *structurally* running into that same problem and that your way of solving it does not really address its recurrent nature.

Second, in the case of patterns of avoidance or denial, the way in which the pattern might prevent a person from solving a problem is by preventing them from recognizing that it is a problem in the first place (117). What is important about such cases is that we are not merely declaring some form of behavior to be a problem even though the person themselves do not see it that way, which would beg the question of arbitrariness. Rather, the assumption behind intervention in such cases should be that it is possible for people to learn about the pattern in such a way that they start to *recognize* the problems that they have been avoiding or denying. If that is the case, then the problems are really problems for the person in question, and we can explain *why* it is a problem in relation to their own values, needs, and goals. In some cases, such a breakthrough may include the revision of those values and goals themselves, which shows how a pattern of denial can be an example of a mechanism that undermines the existential cycle of reflection upon one’s final ends^12^.

In extreme cases, such a pattern which undermines the third cycle of agency may be relevant to a justification of mental health care when people do not recognize that they need help at all. Nevertheless, it should be clear that merely for a practitioner to have good reasons for believing that someone is mistaken about what their problems are does not suffice for this. Even if we can understand values and goals as something that people can be mistaken about, then people have a right to make their own mistakes. Therefore, justification of health care when the person in question disagrees with that justification will have to include the severe effects of these mistakes, perhaps in terms of the suffering of others. A further exploration of this issue deserves a separate essay.

Finally, note that our proposal can also accommodate the opposite scenario, where a person believes they are suffering from a problem-sustaining pattern (or a mental disorder) that requires a mental health care intervention when in fact they do not. This can be discovered in different ways: perhaps they stumble upon a solution to their problem that works, perhaps they discover their problem is not sustained by a mental health issue, or perhaps they discover that it is not really a problem at all, or that there is really a different problem, one that they *can* solve. Sometimes this might happen as a result of a diagnostic procedure together with a practitioner, for example by making a case formulation that provides sufficient insight for the person to no longer require further interventions. In other cases a person might have such a breakthrough on their own or in interaction with people from their social circle. And in some cases a person might never revise their values or beliefs and keep attributing a problem to themselves that their mental health care practitioner will not recognize^13^.

### 4.4 Taking stock

We have now seen how the notion of problem-sustainment offers a generic rationale for providing mental healthcare that can be applied in various kinds of scenarios. In each of these types of scenarios, the concept of problem-sustainment is meant to explain *why* people require mental healthcare. As we already mentioned in section 4.1, that does not rule out political decision-making about how to allocate healthcare resources. When interventions are expensive, the cost of helping needs to be weighed against the cost of not helping. The concept of problem-sustainment explains what that cost of not helping would be. Furthermore, when resources are scarce, it is a political judgment to determine who needs those resources the most. As we shall argue in the next section, problem-sustaining patterns exist in degrees: the extent to which a pattern is undermining problem-solving, and the extent to which a problem recurs or persists, can be less or more. Resource allocation may be decided both in terms of the severity of actual patterns and the severity of possible future patterns that could be prevented by timely intervention. In this manner, the concept of problem-sustaining patterns retains the desirable feature of the established concept that it helps to justify who needs mental healthcare^14^.

The proposed concept even improves upon this feature in the light of the aforementioned arbitrariness of established criteria. The proposed criterion that there is a pattern that sustains a problem in such a way that a person cannot solve the problem without professional help immediately explains why professional help is needed, as opposed to criteria such as a symptom being dysfunctional in evolutionary terms, or a duration of grief being regarded as normal or abnormal by the standards of society. Of course, this only works if knowledge of such patterns is possible, and if there are forms of professional help that are likely to break such patterns. This brings us to the considerations of ontological and empirical soundness, which we shall address in the next section.

The proposed concept also retains the feature of providing recognition of hardship. Problem-sustaining patterns typically explain why the persistence of problems has not been for a lack of trying, why coping strategies may have placed a huge burden on a person’s energy without them fully realizing this, or how sustained conflicts might be the result of well-intended but counter-productive social interactions. Finally, compared to the established concept, problem-sustaining patterns allow more emphasis to be placed on social context and environmental factors that may put someone at a severe disadvantage, without having to resort to the attribution of an individual disorder in order to proceed with a plan for helping a person deal with such challenges.

In this manner, we can provide recognition without having to pathologize diversity, which was one of the flaws that we discussed regarding the established practice. This is also one of those issues where metaphysical, scientific, and normative issues are intertwined. In the next section, we will dive a bit deeper into the reality of multi-level patterns of bio-psycho-social interaction. While we believe there are scientific reasons for explaining problem-sustainment in terms of complex multi-level interactions rather than in terms of independent dysfunctions, we will also argue that this leads to a picture that avoids unnecessary pathologization of human diversity.

Of course, in some cases most of the factors that sustain a problem may be of an individual nature, and in some cases they may involve some form of pathology or dysfunction. To the extent that a problem-sustaining pattern is an individual matter, we believe the concept of such a pattern is still less prone to stigma than the established concept of an individual disorder. First of all, the proposed concept emphasizes the everyday problems that people grapple with, problems that in many cases most people would be able to understand and empathize with. Second, as we shall see in the next section, many of the mechanisms which sustain these problems manifest themselves gradually as stronger or weaker patterns in people’s lives. Patterns of inactivity, for example, not only affect people with all sorts of problems and symptoms, but affect healthy people as well, in various degrees. By contrast, psychopathology has long been viewed as a study of various abnormal behaviors, and the approach to the demarcation problem which has sought to demarcate everyday problems from abnormal problems invites the othering of people who need mental healthcare.

## 5 Patterns of dynamic interaction

Let us now turn to the ‘patterns’ part of the concept of problem-sustaining patterns. This part is mostly intended to address the considerations of ontological and empirical soundness from section 2.2, while it also follows up on some of the points mentioned in the section above about clinical and moral normativity.

Recall once again our overall definition of a problem-sustaining pattern as a ‘pattern of dynamic interaction between biological, psychological, and/or social factors that persistently or recurrently counteracts or undermines everyday problem-solving activities’. In the previous section we have articulated the second half of this definition. While doing so, we have already made use of the notions from the first half: we have loosely talked about ‘patterns’ and we have discussed examples of how biological, psychological, and social factors can play a role in the sustainment of a problem. In this section we will explicate the first half of our definition, ‘a pattern of dynamic interaction between biological, psychological, and/or social factors,’ in further detail.

At the end of section 2.2 we stated the aim of formulating an interpretivist account of diagnosis that would accommodate both realist and constructive intuitions. In section 5.1 we will discuss how the theory of ‘real patterns’ has been used in the philosophy of science and mind to account for various phenomena that are gradually present in the world. In section 5.2 we shall apply these ideas to the domain of mental health. We will argue that this leads to a form of interpretivism about problem-sustaining patterns that avoids the flaws of reification that have plagued the established concept of mental disorders. Finally, in section 5.3 we will discuss how the science of mental health is moving toward holistic and network-oriented approaches based on dynamic interactions between factors, and how patterns of such interactions can explain the sustainment of problems without having to presuppose underlying dysfunctions.

### 5.1 The ontology of patterns

We believe that the concept of a ‘real pattern’ (120–122) may help us to address the challenges of reification. Before we can explain how, let us first discuss what real patterns are and how this concept has been applied in the literature before.

The original idea was proposed by Dennett as part of an interpretivist approach to intentional attitudes, such as beliefs, desires, and intentions (120, 123). According to interpretivism, we can understand intentional attitudes by understanding the conditions under which intentional attitude ascriptions help us explain and predict the actions of the person that the attitudes are being ascribed to, without having to reify the constructs from our ascription language as a kind of entities in the mind of that person (123–125).

One of the questions concerning interpretivism is whether it leads to instrumentalism, the view that intentional attitude ascriptions are merely pragmatic devices that help us predict behavior, without giving us a reason to believe in the reality of those attitudes. Dennett argued against instrumentalism on the grounds that the intentional stance, from which we ascribe attitudes to people, makes certain patterns visible in behavior that are not visible in any other way. The key to Dennett’s argument is that these patterns enable very efficient predictions of behavior, and that even if we would be able to explain and predict the behavior of a person in purely biological and behavioral terms, we would not be able to explain *in those terms* why the intentional stance is that much more efficient at predicting the same behavior. Furthermore, we can reason about real patterns counterfactually in ways that give them genuine explanatory power: intentional stance predictions do not merely latch onto contingent correlations.

At the same time, Dennett argued that these patterns are not new things in any dualist sense, that they are merely present in the behavior of a person, and that their existence is typically gradual, since the intentional stance produces an idealized simplification of the underlying reality. In this manner, Dennett attempted to defend a ‘mild realism’ of intentional attitudes, a middle ground between instrumentalism on the one hand, and the kind of realism that would reify intentional attitudes on the other hand.

Dennett’s approach has inspired a plethora of accounts that leverage the idea of a real pattern in different ways. Some philosophers have argued that the patterns which explain successful intentional attitude ascriptions are not patterns in mere behavior, but patterns in underlying affective processes and empathic interactions (125, 126). Some have focused on what is only a first step in Dennett’s argument, that nature is full of real patterns, and developed detailed accounts of various natural phenomena, from chemical bonds to biological species, as patterns that we can be mild realists about (121, 127–129). There are now different mathematical proposals for the criteria a pattern must satisfy, in terms of algorithmic complexity, in order for it to count as a real pattern (121, 130, 131). There are applications to more complex forms of human action and intentionality, such as valuing (126), selfhood (132), collective intentionality (133), plural agency (134), and microeconomics (135). And there have been some applications in the context of psychopathology, which bear some resemblance to our proposal (132, 136–138)^15^.

In our view, these different approaches are part of an exciting and growing movement in the philosophy of science that helps us understand what the special sciences are about, and which we think is applicable to the science of mental health as well. At the same time, we recognize that the intricacies of the different approaches within this movement are speculative, and discussing them in detail will be beyond the scope of this article. Instead, we will proceed by articulating a number of features of real patterns that most of these accounts agree on, and which seem intuitively correct about certain phenomena that we can easily understand as patterns, such as weather systems like storms and tornados, economic events like crises and inflation, and fluid dynamics phenomena like convection cells and surface waves. In each case, the relevant phenomenon is a *pattern* because it involves regularities or symmetries which are captured by an idealized model that we can use, even though it merely approximates any dataset we may have about this phenomenon.

What makes these patterns *real* is that algorithms that make use of the pattern will be more efficient in compressing such a dataset than algorithms that ignore it. To the extent that the data are accurate, this efficiency should come as a surprise unless we assume that the pattern is *really there*. This idea is perhaps the most metaphysical and speculative part of our proposal. Nevertheless, in the case where we are analyzing data involving tornados in the air, for example, or surface waves on the water, assuming that there are patterns in our data about such phenomena that correspond to *real patterns in the air and on the water* seems very plausible. Few people would deny the existence of tornados, perhaps because they are easy to recognize. Other patterns may require more training or technique to be recognized, such as radio waves in an electromagnetic field or patterns in financial transactions on the stock market. But it can be argued that those patterns are nevertheless also really there, if they allow us to understand the data, and correctly predict further data, in ways that would not be possible if we had not recognized those patterns.

**Figure 1:**
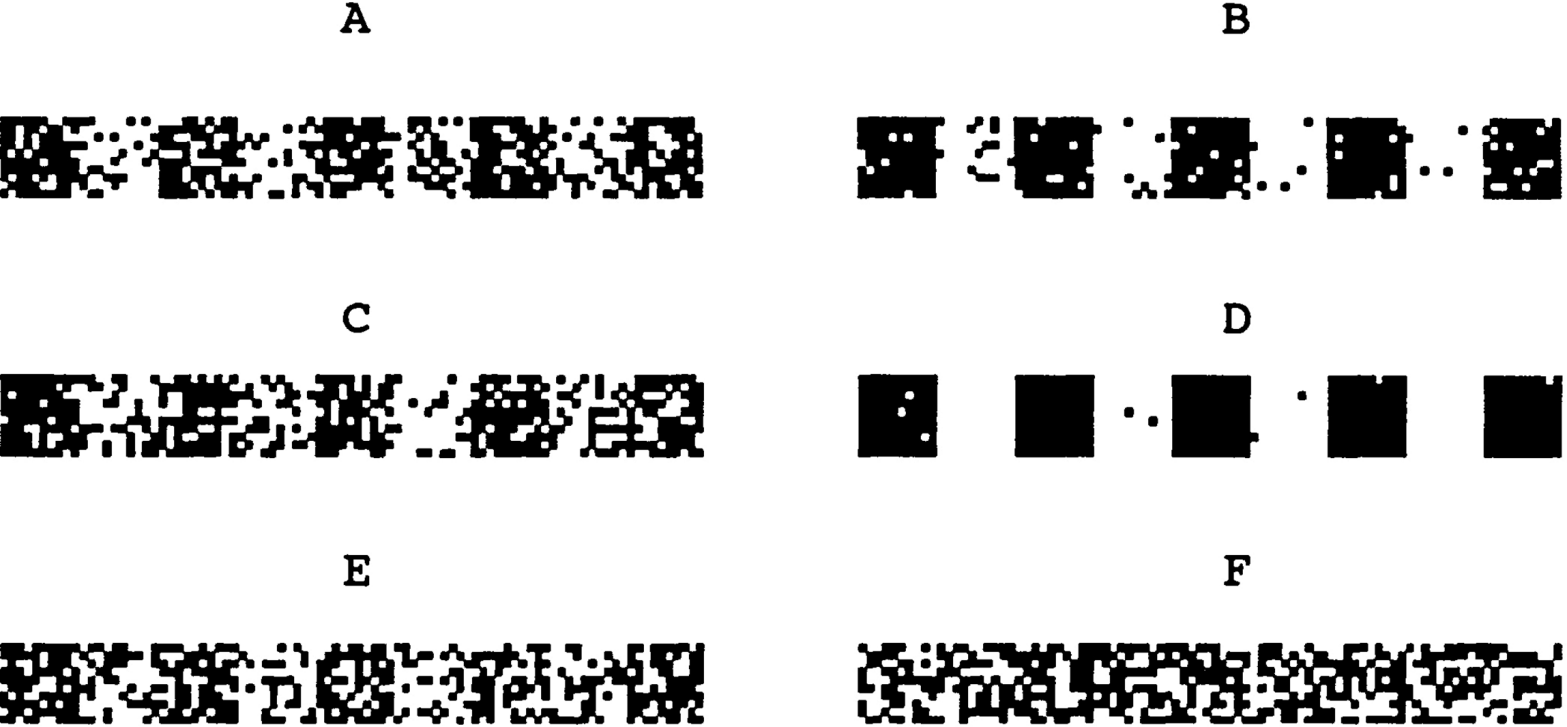
In this example a bar code pattern is affected by different noise ratios. Panels **(A–E)** contain patterns of varying strength. In panel **(F)** the strength is reduced to zero and nothing of the pattern remains. Reproduced with permission from Figure 1, Dennett DC. Real patterns. Journal of Philosophy. [(120), p. 31].

One of the interesting features of patterns is that they can exist to certain *degrees*. **Figure 1** gives us a very simple illustration of this: the pattern is stronger in some of the datasets and weaker in others. There are two reasons why patterns may exist gradually in a dataset. The first reason is when the method of measurement has introduced *noise* in our data. For example, noise in an analog recording can be caused by interference and the quality of the equipment, while noise in a digital file can be the result of bitrot or network errors. In such cases, there is an original signal that is represented by the data, but the data deviates from the signal as a result of the noise. However, there is also a second reason why a pattern might not be perfectly present in the data, and that is when that pattern was not perfectly present in the real world to begin with. Consider the pattern of economic inflation: this pattern is present when prices of goods and services are increasing, but it does not mean that every price of every product will increase. The hypothesis that there is inflation explains a certain amount of price behavior, but not all of it. Or consider a wave on the surface of the ocean, near the beach: we can see a wave on the water and recognize the sine wave shape even though the water will not *exactly* have that shape because of other waves and other things occurring. The idea that there is just one big wave that we’re seeing, and that it has a sine wave shape, is a simplified model that captures a real pattern that is, to some degree, present in the water.

Philosophically, real patterns are a helpful ontological concept because they allow us to understand, both intuitively and mathematically, how certain phenomena can really exist without being ‘thing-like’ in certain respects. For our purposes, the following aspects will be relevant.

First and most obviously, patterns can exist without being fully present, as opposed to material objects like a rock or a chair, which are either there or they are not (quantum superpositions aside). This makes patterns a suitable ontological referent for phenomena that we can only understand through simplified idealizations.

Second, patterns do not exist in their own right, but are only present in a medium, such as a storm in the air, or a wave in the water, or an economic pattern in the behavior of people. Ontologically, patterns add no substance of their own to the world, there is ‘no addition of being’ [(139), p. 117] to the water that the wave exists in, or the air that the storm exists in.

Third, different patterns can *overlap* in the same medium in the same place. Multiple waves can overlap on the surface of the water, or different economic patterns can explain the same price changes. Sometimes pattern *A* results in noise relative to pattern *B*, such as when a small wave creates imperfections in the shape of a larger wave. Sometimes different patterns can create a resulting pattern of interference. And sometimes the same regularities in a medium can simply be explained with different models, which recognize patterns that are similar to each other, such as when we can approximate a collection of data points using different mathematical curves. These overlapping models may vary in complexity and accuracy. Real patterns can be identified at different levels of simplification, providing us with trade-offs between usability and accuracy that may be decided upon pragmatically [(120), p. 36].

Fourth, patterns often have fuzzy spatial and/or temporal boundaries, and there may sometimes be no fact of the matter about how to count or individuate them. One of the ways in which a storm is not a ‘thing’ is that it must be to some extent indeterminate when and where a storm begins and ends. Furthermore, if there are multiple local air pressure minima in an overall low-pressure area, do we count this as multiple depressions or as one big depression? The answer seems arbitrary while the facts are already clear.

Fifth, the interpretation of a pattern becomes a reflexive process when the interpreter is part of that which is being interpreted. This applies specifically to psychological, social, and economic patterns, when we are trying to make sense of our own behavior and motivations, either collectively or individually. Widely shared beliefs about the direction of the economy, for example, can sometimes influence people’s choices in such a way that the economy is pushed further into that direction as a result, for better or worse.

At the individual level there is a similar kind of ‘snowball effect’ (140) with regard to how our self-adopted values or goals may gain weight and shape our lives in such a way that we acquire more reasons to pursue them. Consider again the existential cycle of agency that we discussed in section 4.2. On the one hand, a person may sometimes discover that their self-ascriptions about what they want or value are really misguided and a poor fit with what they need and who they are. In terms of patterns, we might say that such self-ascriptions fail to recognize a pattern in someone’s various dispositions (126). On the other hand, we have seen that patterns admit plurality and indeterminacy. In some cases, two different self-interpretations might capture equally strong patterns in someone’s volitional personality, but once one of those has been adopted and acted upon, it strengthens the pattern in question, as the person becomes more committed and attuned to the chosen path in life. Self-interpretation is therefore neither fully recognitional nor fully constructive, but a reflexive process that has both aspects.

### 5.2 Application to mental health

Let us now apply this concept of real patterns to the case of mental health. According to our definition of problem-sustaining patterns, these would be ‘patterns of dynamic interaction between biological, psychological, and/or social factors’. Thus, the medium for these patterns would be the realm of biological, psychological, and social activity. Problem-sustaining patterns may be present in someone’s interaction with their spouse, or in the interaction between their feelings of reward, activity in certain parts of their prefrontal cortex, and their work habits^16^. Let us first review how the aforementioned characteristics of the concept of real patterns might help us avoid the three forms of reification discussed in section 2.2. Then we will consider how the realist and constructivist aspects of interpretivism combine in the case of diagnostics.

The first form of reification we identified was the assumption that there are independent diseases or dysfunctions behind the different syndromes, even when a single person has been diagnosed with multiple syndromes. But if problems are sustained by real patterns, then the idea that such patterns can overlap, and that different models can capture similar regularities in our data in different ways, may help us understand what it is that syndromes like BPD or ADHD manage to capture, and why it is that they appear to show such comorbidity. This is not because people who are diagnosed with both have two separate diseases, but it is because there are multiple patterns that involve symptoms like impulsive behavior or thrill-seeking behavior, and in the lives of some people many of these patterns play a role to some degree.

The second form of reification was the assumption that a disorder is something a person either has or does not have. But the *gradual* nature of patterns makes them fundamentally different from that. A pattern of interaction between negative thoughts and expectations, social inactivity, and feelings of sadness, for example, might affect many of us a little bit when we are down, while it may also be a core mechanism sustaining severe depression in the case of people who really need professional help. Counting how many people are depressed in the latter sense, or determining when the former turns into the latter, is to some extent undetermined, like in the example of local air pressure minima (depressions in the meteorological sense).

The third form of reification was a confusion of the idealized models that we use to explain mental health problems with the actual mechanisms behind those problems. Recognizing this difference is what makes mild realism mild: the ascribed interpretation is an idealization which simplifies the underlying noisy reality. Even if we may never know the full causal story behind a single sleepless night, what we do know is that there are many patterns which influence our ability to sleep positively and negatively, such that none of these patterns are likely to predict every sleepless or sleepful night. Nevertheless, in the case where the sleepless nights occur frequently, there are typically a number of ways that we can figure out which patterns might be playing a leading role, from investigating what happens when a person changes their habits and routines, to performing observations or EEG measurements in sleeping labs.

We believe that by explicitly talking about patterns and developing pattern-like models and visualizations, we can help people realize that the models are merely gradually realized by a far more complicated reality, much like in the case where we visualize the model of a storm or waves on the water surface.

By contrast, treating disorders as if they are caused by disease entities invites people to expect an unrealistic degree of accuracy and determinacy from psychiatric diagnosis. Of course, such expectations are not based on definitions or conclusions in either the DSM-5 and ICD-11 or scientific research regarding the syndromes defined in them. However, they follow from the associations that people have with the language of disorders, and from the application of the medical model in the established practice of mental healthcare.

While an alternative practice that explicates and visualizes pattern-like features requires further design work to flesh out, we have some anecdotal information indicating that people, including clients, professionals, and others, seem to like the language of *patterns*. In many cases, people appear to prefer the terminology of ‘being stuck in a pattern’, for example, to that of ‘having a disorder’^17^. And in the case of problems that are sustained over a longer period of time and across different situations, talking about ‘pervasive and persistent patterns’ may be preferred over ‘personality disorders’^18^.

Let us now consider how this mildly realistic approach may be combined with insights from constructivism. We have seen how interpretivism allows for interpretation to become a reflexive process, in which the interpreter, and the models applied by the interpreter, become part of the pattern that is being interpreted.

In this way, social, clinical and scientific constructs may feed back into problem sustaining patterns, for good or ill. At the individual level, this may for example occur when a client and a practitioner try to analyze the relations between various factors that sustain a problem. Some of these factors, such as certain thoughts or feelings, may be articulated more explicitly than they had been before. A sentence may be used to explicate a certain fear or negative expectation, and such a sentence may explain to some extent what someone has been feeling and what has been driving their behavior. But as a result of this explication, the next time that this person has this feeling or finds themselves in a situation where such behavior occurs, the feeling may become more aware and more shaped according to that framing. Even when this might lead to a temporary sharpening or perhaps increase of symptoms, it may also make them more tractable, especially if the way the symptoms are framed corresponds to the ways in which they are also addressed in the recommended therapeutic intervention.

A different type of case is where the client’s unawareness of the problem-sustaining pattern is such an important part of the pattern, for example because of the counterproductive way in which someone keeps trying to solve their problem, that the mere understanding of such a pattern may cause it to be largely diminished, as it removes the motivation for the person to behave the way they did. Interpretative models can also have adverse effects, for example when the applied constructs inadvertently strengthen the interaction patterns that sustain a client’s problems, as in the case of stigmatization due to misdiagnosis.

At the collective level, these kinds of interactions between problem sustaining patterns and our understanding of them is reminiscent of what Hacking termed ‘looping effects’ (141, 142). According to Hacking, our diagnostic practices may change the people diagnosed, by inducing new ways of sense-making enabled by the diagnostic classification system. Tracking such changes in experience and behavior may require adjustments to the classification system, which in turn may lead to further changes in the people classified. Thus, a feedback loop is created between the classification practice and the people classified, turning mental disorders into moving targets.

We believe such looping effects are real phenomena in mental health care. Looping effects may gradually shift diagnostic classifications, but they may also stabilize them into a local minimum. Radical phase shifts may occur in certain areas of mental state space of human populations due to the introduction of new interpretative models in the context of wider societal, political or economic change. In a sense, our project is premised on the existence of such looping effects: reframing mental health problems in terms of problem sustaining patterns is aimed, among other things, at changing our sense-making practice in mental health care—our ways of interaction, (diagnostic) (self-)interpretation, therapeutic interventions, etc.—so as to nudge the dynamics of people’s problem sustaining interaction patterns in a more favorable direction.

### 5.3 From dysfunctions to dynamic interactions

In section 2.2 we have discussed the feature of evidence-based treatment advice, and the flaws of poor external validity and reification of syndromes. We have just argued how the idea of real patterns may help address the flaws of reification. However, our definition of a problem-sustaining pattern mentions a specific type of pattern: a ‘pattern of dynamic interaction between biological, psychological, and/or social factors.’ Let us now shift our focus to this notion of *dynamic interaction* between these various types of factors. This notion is meant to reflect a shift in the science of mental health that may improve upon the poor validity of established systems of classification while allowing better tailored treatment advice in individual cases.

The idea of dynamic interaction between multiple factors may be contrasted with the idea of a singular (set of) dysfunction(s) as the cause of a mental health problem. We have already noted how a person who has been diagnosed with multiple syndromes need not have separate underlying dysfunctions for each of those syndromes. Conversely, even when a person has been diagnosed with only one syndrome in current psychiatric practice, we should be skeptical about the idea that a well-defined (set of) underlying dysfunction(s) is causing their symptoms. As we discussed in section 2.2, the search for such dysfunctions has so far been largely unsuccessful. Instead, as we have mentioned in section 3.2, there is a trend toward holistic, complex system approaches that emphasize the interaction between factors to explain mental health issues, rather than any intrinsic dysfunctionality of the factors themselves^19^.

Of course, in order for the interaction between these factors to constitute a problem-sustaining pattern, one or more of these factors do have to pose a problem for the person or people in question, in line with our analysis of problem-solving agency from section 4.2 above. However, it is the whole pattern of interaction between all these factors that makes it difficult or impossible for the person or people in question to solve these problems on their own. In this manner, the idea of problem-sustainment can be combined with holistic approaches to mental health.

**Figure 2:**
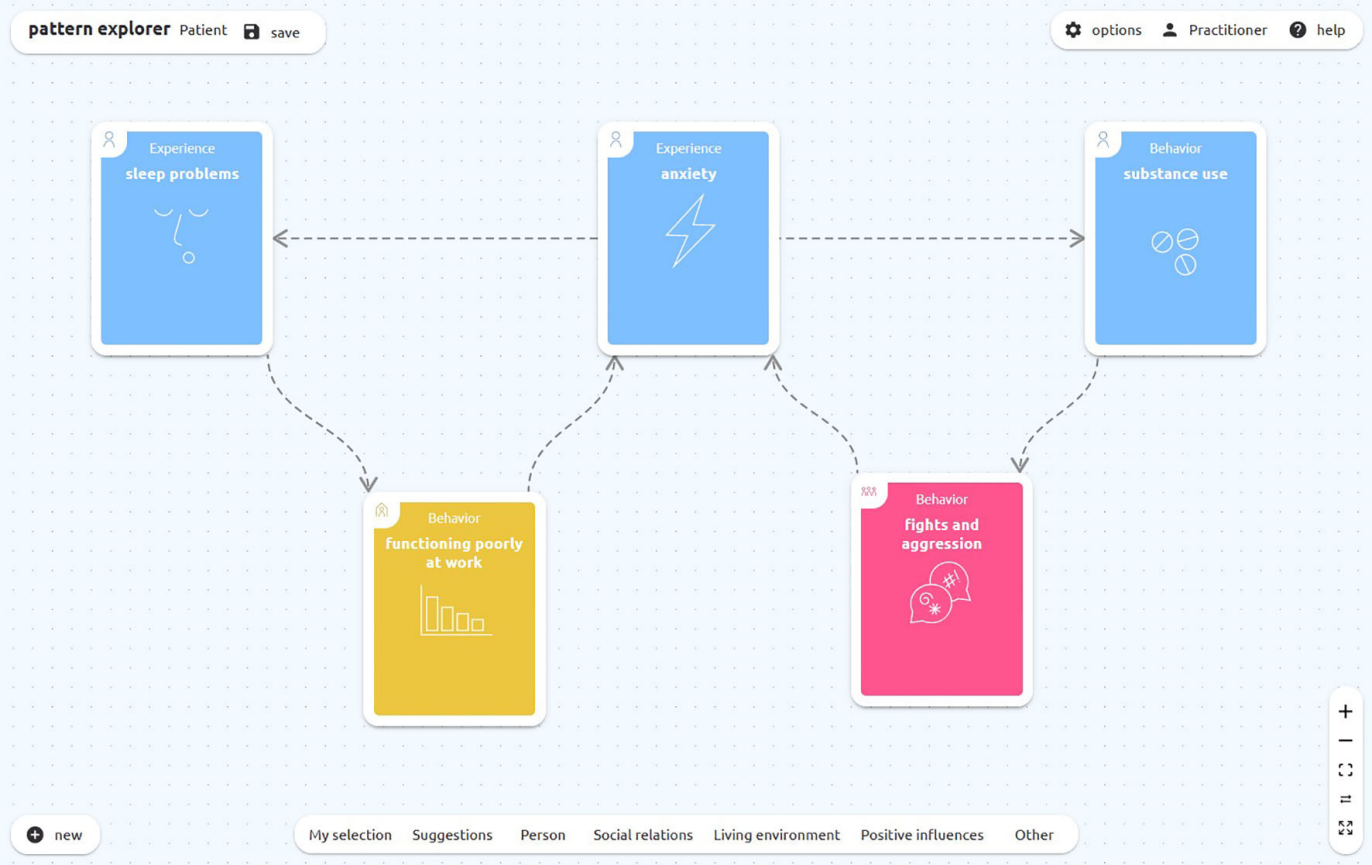
Example of a simple model of two feedback loops in the *Pattern Explorer*, an e-health tool we are developing to facilitate interactive cocreation while at the same time generating data about the case formulations that people make.

According to complex systems theory, these interactions are typically nonlinear, such that feedback loops or equilibria emerge (see **Figure 2**). This means that the patterns that sustain our problems have a way of becoming part of our lives in the same way that ‘healthy’ equilibria, such as our valued social customs or work habits become part of our lives. Mental healthcare may thus be viewed as a form of assisted equilibrium-shifting or phase transition (40, 68), where the goal is often not merely to disrupt the problem-sustaining equilibrium, but also to establish and strengthen a new equilibrium that facilitates the client’s values and plans.

To understand how such an equilibrium-shift might be brought about, one needs to know which factors or interactions within the pattern might be the most promising entry points to intervene upon, so to speak, and which interventions would be most likely to succeed in the particular circumstances of a person or persons. In section 2.2 we have noted that although the established paradigm of syndrome-specific treatment protocols has brought valuable knowledge of the effectivity of various interventions that we do not want to lose, that knowledge is at the same time difficult to apply in individual cases due to the poor external validity and heterogenous nature of the syndromes in question. Furthermore, when research into different interventions has demonstrated similar effect sizes for the same syndrome, or for different syndromes that a particular patient has both been diagnosed with, that does not mean each will work equally well for that patient.

Hoffman and Hayes have argued that this problem cannot be overcome merely by switching to a transdiagnostic symptom-specific paradigm (39). Instead, they argue for a focus on *processes of change*. The new standard of evidence-based practice, in their view, should require for interventions to be based on testable models and theories about processes of change and the conditions under which certain processes could be activated by certain interventions. Moderation and mediation studies can help to provide evidence for such models and foster evidence-based decision-making on the basis of an analysis of a problem-sustaining pattern instead of merely a patient conforming to a classification. Thus, we can think of these processes of change as routes by means of which the aforementioned equilibrium shifts can be achieved.

According to our proposal, the pattern of dynamic interaction that sustains a problem can be between biological, psychological, and social factors. This general idea goes back to the ‘Bio-Psycho-Social’ (BPS) model of health (143). In our view, biological, psychological, and social factors can all influence each other. In section 4.3 we have already discussed some examples of how interactions between these various types of factors can sustain problems. Let us now elaborate a bit more on what we mean when we talk about these different types of factors.

For starters, along the lines of our interpretivist ontology of ‘mild realism’, the phrase ‘biological, psychological, and/or social’ is meant to include a range of perspectives or ‘stances’ from which relevant factors that contribute to a pattern may be brought into view (121). Many clinical approaches and individual professionals tend to have a dominant perspective from which they are most likely to identify elements of a pattern, with the risk that other relevant factors are being overlooked. We want to stimulate the clinical practice to adopt a wide view. Of course, this does not mean that every individual case should be assessed from all perspectives. But our practice of mental healthcare should be able to adopt each of these perspectives when they are relevant and explore the interrelations between them in order to understand how a problem is being sustained.

Note also that the idea is not that there are exactly *three* stances, the biological, the psychological, and the social, nor that there are clear distinctions between them. Biology alone includes many subdisciplines that can be relevant to mental health, from genetics and neuroscience to ecology, which can adopt different stances and model the world in different ways and at different scales. The same is true for subdisciplines of cognitive and social science. Some factors may be difficult to classify into one or another discipline. If a person is tired, is that a psychological or a biological property? Perhaps we can make further distinctions but the point of recognizing patterns through idealized models is that perhaps in many cases we don’t have to. Thus, when we speak of ‘biological, psychological, and/or social factors’ this is a shorthand for saying that factors may be relevant that can be brought into view by combining this whole range of subdisciplines.

Furthermore, in some cases where the social perspective is relevant, the shift from dysfunctions to dynamic interactions allows us to identify certain patterns of *interpersonal* interaction that sustain problems in such a way that they cannot be attributed to the mental health of merely one of the interacting persons (as systems therapy has long acknowledged). Thus, problem-sustaining patterns range from intrapersonal interactions to thoroughly interpersonal dynamics.

On the former end of the spectrum are problems that are being sustained by interactions between individual factors, like biological factors and personal habits, such that a client can focus mainly on individual work to break the pattern. Note that even in such cases, it does not really make sense to say that the problem-sustaining pattern exists ‘within’ the person, or that the person ‘has’ a pattern that sustains a problem. Ultimately, all behavior is interaction between a person and their environment, and even if the client can achieve a change on their own end, so to speak, the pattern is best understood as a situation they are ‘stuck’ in rather than a disorder that they ‘have’.

In the middle of the spectrum are cases where a client’s social environment is relevant to such an extent that there needs to be a match or attunement between what works for the client and what is expected from the environment, but where the primary responsibility to address this matter may still be considered to lie with the individual client.

Finally, at the interpersonal end of the spectrum are patterns among couples, families, departments, or even society at large, such that the patterns in question cannot be addressed without some form of collective responsibility for action. In the case where the pattern exists at the level of a family, for example, interventions like couples therapy or systems therapy make more sense than individual treatment, but these may still be considered within the domain of mental health care. When we consider larger groups of people, there are patterns that affect the mental health of individuals even though the patterns themselves cannot be broken by the interventions that mental health institutions typically offer, such as workplace bullying within a team or department at a company, or patterns of institutional racism, homophobia and sexism within society at large. Thus, even though some individuals might seek mental healthcare to deal with traumatic effects of homophobia, the fight against homophobia itself is a social and political struggle.

It is common, though not uncontroversial, to think of the various systems and subsystems that may be brought into view through these various perspectives as organized into different levels (47, 81, 136, 144). Presumably, a social conflict at work is a higher-level property, which might increase with the ways people respond to one another, and which influences their cortisol levels or blood pressure or some other presumably lower-level properties. Should we think of the social conflict as a high level cause of the rise in cortisol and blood pressure, or can we somehow analyze the social conflict into lower level events that cause these effects? Or is the increase in blood pressure itself a part of the social conflict? It is not our intention to defend a specific metaphysical account of inter-level causation, except that it should be possible in the trivial sense of the above example. While these metaphysical questions are interesting and a fully articulated account might enrich our understanding of problem-sustaining patterns, it would be strange if adoption of the very idea of problem-sustaining patterns would have to turn on whether someone would prefer, say, an emergence theory of interlevel relations over a supervenience theory.

What we should note however is that any factors that are attributed to a person or situation as part of a problem-sustaining pattern are conceptualized as part of the *model* of that pattern. In the context of such a model, a phrase like ‘social conflict at work’ is just another idealization. It is not an attempt to cut nature at the higher-order joints or to specify an event that is causally fully distinct from other factors within the model. If we are not to reify the entire model, then neither should we reify the levels implicit in our model, or expect that the different factors we attribute are fully distinct from each other. In other words, our proposal implies mild realism about interlevel causal relations within models of patterns, without subscribing to any further metaphysical theory about causality in the actual world^20^.

The ontological gist of our proposal shows some resemblance with Kendler et al.’s ‘mechanistic property cluster view’ of psychiatric disorders (47). According to this view, mental disorder kinds are to be understood as relatively stable clusters of properties identified at multiple levels (molecular, neurobiological, psychological, social/environmental, etc.). The ‘kindness’ of psychiatric disorders, on their view, is not produced by a defining essence or dysfunction, but rather by cross-level mechanisms that explain why these properties tend to cluster together through the course of illness trajectories. Our proposal both refines and reframes this idea of psychiatric disorder kinds in terms of a typology of problem-sustaining patterns. The notion of cross-level mechanisms explaining the clustering of multi-level properties through time, is in line with our understanding of the cross-level dynamics of problem sustainment.

## 6 Conclusion

In this paper we have approached the established concept of mental disorders as an artifact with certain features and flaws. We have proposed to redesign it into a concept of problem-sustaining patterns. This revised concept is meant to preserve the features of the established concept while improving upon its flaws.

Clinically and ethically, the most important features of the concept of mental disorders are the recognition that diagnosis can provide and access to certain forms of healthcare. However, this presupposes a philosophical justification of why some people require such recognition and care for their problems, while others do not. This is not merely a theoretical issue, but a normative one too, because it is ultimately based on a societal agreement on key values informed by currently available scientific evidence. Ignorance of this normative dimension opens the door to all kinds of stigmatizing and iatrogenic effects. We have argued that established justifications of mental disorders rely too strongly on societal standards of functioning as well as assumptions about the intrinsic dysfunctionality of symptoms or the presupposed latent factors underlying those symptoms. Instead, the concept of problem-sustaining patterns is based on an agential approach, which shifts our focus from the nature of the problems that people have to the nature of the ways in which those problems are being sustained. This reduces stigmatization because it normalizes all problems that people struggle with, regardless of whether they need help, and it does not pathologize factors that only contribute to the sustainment of problems in the context of specific patterns. Furthermore, it clarifies the nature of clinical expertise, which is about the ways in which problems may be sustained. And finally, it provides a specific goal for mental healthcare (in collaboration with other services): to break those patterns that sustain problems.

An important scientific feature of the concept of mental disorders is that it facilitates an evidence-based paradigm that has yielded valuable knowledge about the effectiveness of various interventions. However, the application of this concept in practice has lead to a *reification* of mental health problems. By thinking in terms of *patterns* we can instead do justice to the gradual, fuzzy, and complicated reality that sustains problems, while recognizing the idealizing and simplifying character of the models we use to capture such patterns. A further scientific and ontological flaw in the established concept is the assumption that there must be some specific, circumscribable (psychological, biological, developmental) dysfunction underlying mental disorder, which has led to a decades long search that has not provided satisfactory confirmations of this assumption^21^. Instead, we propose a causal model of patterns of dynamic interaction between various factors, which is supported by promising developments in network theory and complex systems theory. Thus, instead of diagnosing disorders or eliminating diagnosis altogether, we propose that diagnosis should be focused on identifying the complex interactions that sustain problems, and insofar possible, the mechanisms that explain those interactions and the processes of change that might help a person break out of those interactions.

Our concept of problem-sustaining patterns is still work in progress and several challenges need to be addressed. The first challenge concerns cases of problem sustainment where the person in question does not consider their (pattern of) action to constitute a problem or problem-sustaining pattern, but where we do have good reason to regard it as such and also to regard the problem-sustaining pattern as a proper target for mental healthcare. We have sketched how the model of agency behind our proposal, which includes an existential cycle of value revision, makes it possible to attribute problem-sustaining patterns that undermine this aspect of a person’s agency. The idea is that we can explain their problem in terms of the values they would adopt if not for this pattern, rather than in terms of values of the practitioner or society that the person in question might have no reason to adopt. The challenge is whether this type of justification would be sufficient to account for a spectrum that ranges from merely directive and persuasive forms of therapy aimed toward changes in motivation to forms of involuntary care in more extreme cases where a person poses a danger to others or themselves^22^.

A second, related, challenge is to further explain what makes a revision of a person’s values and goals (the third cycle of agency) to genuinely reflect values and goals *of their own* (rather than of e.g., society, their parents, their therapist). This involves detailed philosophical analysis of what it means to have normative reasons of one’s own [e.g. (108–110)].

Thirdly, in cases where client and practitioner do agree about which problems or symptoms are the reason to start mental health care, the challenge is to show for a wide range of mental health problems whether the model of problem-sustaining patterns would be helpful. In this paper we have tried to discuss various examples to illustrate the proposal. We have also explained how the proposal is largely based upon existing approaches, such as case formulation in cognitive-behavioral therapy, or network analysis in complex systems theory. These approaches do not match very well with the established systems of classification, yet they have been studied extensively with respect to a wide array of mental health issues. This should give some indication of how the concept of problem-sustaining patterns would apply in cases that we have not discussed in this article. Nevertheless, in some cases it might not be obvious what sustaining pattern would play a role in the justification of treatment for a problem, even though intuitively the problem seems to be within the domain of health care.

Obviously, we need to test our conceptual design and show how our proposal can actually improve the science and practice of mental healthcare by reframing our understanding of mental health problems in terms of problem sustaining patterns. Attempts are currently under way to use new diagnostic tools and smart phone apps to help service users understand and manage their mental health issues in terms of (breaking) problem sustaining patterns. And at the meso-level, the Redesigning Psychiatry program is involved in several projects in The Netherlands to reorganize and reallocate mental healthcare services provided by healthcare institutions informed by this new conceptual tool. Meanwhile, other alternative approaches to diagnosis that have already shown promise in practice [e.g. (1, 67, 68, 71, 72).] are closer to the idea of problem-sustaining patterns than the established concept of mental disorders.

These challenges reflect the fact that we are not proposing a conceptual analysis of mental disorder in the sense of providing jointly necessary and sufficient conditions. Rather, our more humble and more realistic aim is to revise the established concept and provide a conceptual tool that enables us to improve mental healthcare.

## Data Availability

The original contributions presented in the study are included in the article/supplementary material. Further inquiries can be directed to the corresponding author.

## Footnotes

1 Cf. other revisionists about mental disorder (1, 2), disease (3–5), and free will (6–8).

2 While this paper focuses on interactions between biological, psychological, and social factors, the proposal might be extended to include other environmental factors, such as air pollution or noise.

3 We refer to the ‘role’ of a concept in a generic sense. It may be understood in functional (9, 10), inferential (11), or practical (12–14) terms, depending on assumptions beyond the scope of this paper.

4 This includes rethinking the place of mental healthcare in our society and its relation to other sectors like education, the work environment, etc.

5 These sessions were planned and organized using the ‘Vision in Design’ methodology (18), and were focused on a wide range of topics including finance, ethics, clinical research, professional roles, resilience, and prevention.

6 For a philosophical defense of normative concepts as artifacts, see (12). For design as mediation, see (19). For design as the transformation of social interaction, see (20).

7 This is not to say that the presence of auditory hallucinations is *sufficient* for a diagnosis of schizophrenia or some other mental disorder in the DSM. According to the DSM-5 definition of mental disorder, ‘A mental disorder is a syndrome characterized by clinically significant disturbance in an individual’s cognition, emotion regulation, or behavior that reflects a dysfunction in the psychological, biological, or developmental processes underlying mental functioning.’ (30, italics added). Our present point, however, is that hearing voices has typically been framed as pathological in modern Western psychiatry and society, something which, if other conditions are met, is part of a disorder according to the DSM.

8 Discussions of reification span a number of philosophical issues and disagreements, some of which are metaphysical, others epistemological, ranging from matters that pertain specifically to how we talk about psychiatric syndromes (55), via issues that concern social and psychological constructs more broadly (57), to the most general questions about our attitudes towards models and idealizations that might equally be raised concerning, say, chemistry or physics (58).

9 With the exception of the very idea of patterns, we try to keep the metaphysical commitments of our proposal concerning the relations between biological, psychological, and social factors to a minimum. We return to this matter in section 5 below.

10 Note that although problems clusters might be associated with certain neurocognitive profiles, this does not entail that the problems are *caused* by these profiles. In line with recent neurodiversity models of dysfunction [e.g. (105)], for example, these problems might be understood as resulting from relational ‘mismatch’ between the needs of neurodivergent individuals and the opportunities the environment affords.

11 Furthermore, as we have already discussed, hearing voices is not that uncommon, and we should refrain from pathologizing it. Rather, it is the interaction between hearing voices and other factors (e.g. salience, stress, paranoid beliefs, beliefs about and attitudes towards the voices, etc.) that typically sustains the types of problems we are discussing here.

12 Does this approach presuppose a form of political liberalism, in the sense that the pursuit of each person’s own values and goals is being promoted? While a thorough response to this question would require an essay in its own right, it should be noted that there is a certain dilemma between libertarian and authoritarian views that we are trying to overcome here (118, 119). Our proposal implies neither the libertarian approach of taking all prior wishes or goals of a client at face value, nor the paternalist approach of imposing the values of the care provider when those prior goals of the client might seem problematic. Instead, we propose that the relevant values that should explain the problems of the client are values that the client should in principle be able to reach by means of the existential cycle of value revision, which is a process that does involve a lot of social context. This intuition should make sense from the perspective of psychotherapy. After all, the client is ultimately the one who has to acquire the motivation to change their life and to embrace a perspective on their issues that allows them to take charge of their life again. How can a psychologist help a person to get there if there is no route from where the client is currently at, towards that destination? Possible counterarguments to this intuition might be, first, that some people have mental health problems that are beyond treatment, or second, that some forms of treatment might change the value system of a person in a manner that does not involve the existential cycle of value revision (e.g. through medication or some other physical intervention on the brain, say). Whether such alternatives would be possible and ethical is a question that requires a discussion elsewhere.

13 One of the reviewers for this article mentions an example of a catholic homosexual person in the 1950s in the US. Based on his values and his beliefs, his attraction to people of the same sex might be experienced as a problem, and if he is not able to make this problem go away, then he might therefore regard his sexual orientation as a problem-sustaining pattern. The point here being that people may not have values of their own that much, as those values are determined to a large extent by their social context. However, the point of the existential cycle of agency is precisely to distinguish between the adopted values of a person and the values that would reflect self-knowledge if adopted. Just because there are social contexts in which it will be difficult or unlikely that a person would challenge the influence from their social context does not mean they might not have good reasons to do so. The analysis of those reasons would depend on meta-ethical assumptions beyond the scope of this article. In practice, we think the sort of view about value revision that we have proposed in section 4.2 above is what a practitioner should focus on in such cases. The mental health professional need not agree that the client’s homosexuality is a problem, nor need they start out by declaring the client’s adopted values to be the problem instead. They can simply agree that the *mismatch* between the adopted values and the feelings of the client pose a problem, and work from there.

14 Note that our concern in this article is justification in a normative sense. This includes the role for political decision-making in our analysis. One reviewer has remarked that the established concept has also served a further political purpose of keeping psychiatry and clinical psychology in business by reifying mental health problems as disorders that society is willing to spend resources on. To what extent would this change if we organize the practice of mental health care around a concept that is less medical, less strictly delineated, and less reflecting of prevailing societal standards of normal behavior? Answering this question may require a dive in critical social and political analysis beyond the scope of this article. However, we do believe that a convincing political argument can be made why investing in the prevention and treatment of problem-sustaining patterns is both beneficial and cost-effective to society.

15 Borsboom et al. (136) appeal to Dennett’s notion of a real pattern specifically in the light of the role of mental content associated with certain factors within their network approach to psychopathology. Thus, they adopt a specific role for real patterns within causal networks in the light of Dennett’s analysis of mental content as real pattern. By contrast, we are proposing to understand the entire network of dynamic interaction as a real pattern, not merely in virtue of the role of mental content or the usage of mental language to identify the psychological factors in the network, but rather in virtue of the fact that the complex causal feedback loops that sustain problems exist themselves as patterns in an even more complex causal reality. For a similar response, see Ross (138).

16 In some cases we may also have to include physical environmental factors as part of this multiscale medium, such as when someone’s work habits involve exposure to toxic substances that affect their psychological functioning.

17 We have been using this language in a number of our design projects and interviewed potential users about how they experienced this. Among these projects were *Bumpy Road* (www.bumpyroad.nl), *Mentaal Lokaal* (www.mentaallokaalwassenaar.nl), and *Patterns of Life* (www.patternsoflife.nl). For example, one interviewee for *Mentaal Lokaal* explained it as follows: ‘I would like to go somewhere and be able to say “I am in this pattern and I would like to work on that” instead of “this is what’s wrong with me”. A motivating vibe.’

18 This preference was expressed by one of the reviewers for this article.

19 We do not mean to completely abolish the term ‘dysfunction’: perhaps a thyroid gland producing insufficient amounts of thyroxine, for example, could reasonably be understood as a dysfunction, and play an important role in the sustainment of a problem, such as a lack of energy. However, then it is still because such a factor contributes to the sustainment of a problem, that the person in question needs help. Furthermore, if none of the factors that together sustain a problem qualifies as a dysfunction, the person would still require help.

20 This is consonant with an interventionist view on causation (145).

21 Although etiology has officially been removed from the DSM since 1980 (with the DSM III), the framing of mental disorders as a ‘clinically significant disturbance in an individual’s cognition, emotion regulation, or behavior *that reflects a dysfunction* in the psychological, biological, or development processes underlying mental functioning’ (30, italics added), has promoted the ontological motivation for the search of underlying (neurobiological, psychological) mechanisms as the common causal pathway for developing mental disorders.

22 As this type of approach would attempt to explain conflict as a result of mental health problems, it may be contrasted with approaches that reason in the opposite direction, and see the practice of attributing disorders more as a response to fundamental conflictual forces in society.
